# Comparative small RNA profiles of beet mosaic virus (BtMV), beet mild yellowing virus (BMYV) and beet yellows virus (BYV) infected *Nicotiana benthamiana* and *Beta vulgaris*

**DOI:** 10.1016/j.virusres.2025.199640

**Published:** 2025-10-10

**Authors:** Dennis Rahenbrock, Mark Varrelmann

**Affiliations:** Institute of Sugar Beet Research, Holtenser Landstraße 77, 37079 Göttingen, Germany

**Keywords:** RNAi, BtMV, BMYV, BYV, Virus-derived small RNA profile, Sugar beet

## Abstract

Plants are constantly challenged by viral pathogens that can limit growth and reduce yield. A key component of the plant innate immunity is RNA silencing, in which viral double-stranded RNA (dsRNA) intermediates are recognised and processed into virus-derived small interfering RNAs (vsiRNAs). These vsiRNAs direct the degradation of viral genomes, thereby restricting infection. Sugar beet (*Beta vulgaris* subsp. *vulgaris*) is a crop of major economic importance, where the virus yellows (VY) complex represents a serious threat to production. Here, we profiled and compared vsiRNAs generated during infection of the natural host plant *B. vulgaris* and the experimental host plant *Nicotiana benthamiana* with three taxonomically distinct viruses: beet yellows virus (BYV, *Closterovirus*), beet mild yellowing virus (BMYV, *Polerovirus*), and beet mosaic virus (BtMV, *Potyvirus*). High-throughput sequencing of small RNAs revealed characteristic size distributions and strand biases that differed among viruses and host species. Comparative analysis highlighted no host plant-specific pattern of vsiRNA accumulation. This comparative approach provides a detailed view of vsiRNA processing and offers novel insights that are not apparent from coverage profiles alone. Distinct vsiRNA hotspots were detected for each viral genome, and these hotspots did not differ between host plants, pinpointing potential target regions for RNA interference-based control approaches. The identification of such regions provides a basis for the design of synthetic dsRNAs that can be applied exogenously as protective sprays, an emerging, non-transgenic strategy to mitigate VY infections, while advancing understanding of vsiRNA biogenesis in sugar beet and *N. benthamiana* in general.

## Highlights

• Plants use RNA silencing to restrict viral infection via vsiRNAs.
• vsiRNAs of BtMV, BMYV and BYV in sugar beet and tobacco were analysed.
• vsiRNA profiles differ by virus type and but not by plant host species.
• vsiRNA hotspots identified target regions for dsRNA sprays for virus control.

## Graphical abstract

**Figure:**
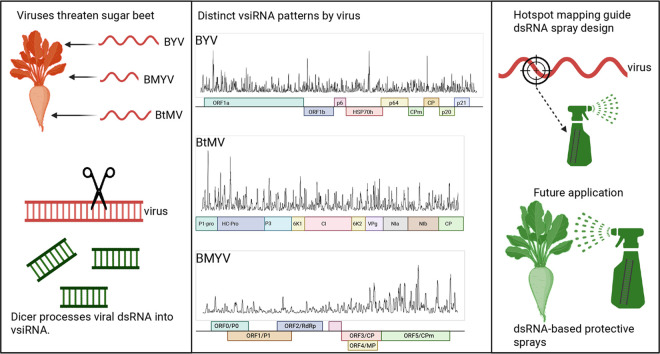

## 1 Introduction

Plants are constantly exposed to viral pathogens that can severely compromise growth and productivity. To defend against these infections, they rely on an innate antiviral mechanism known as RNA silencing, more precisely referred to as post-transcriptional gene silencing (PTGS) or RNA interference (RNAi) (Hamilton and Baulcombe 1999). Central to this pathway is a class of small RNAs (sRNAs), which are generated from viral dsRNA intermediates by RNase III-like enzymes called Dicer-like (DCL) proteins (Bernstein et al., 2001). During viral replication, the viral RNA-dependent RNA polymerase (RdRp) synthesises a complementary negative-strand using the positive-strand RNA as a template, which can create temporary dsRNA intermediates (Ahlquist 2002). Furthermore, viral RNAs can contain imperfect duplex structures in the most folded regions, forming short parts of dsRNA, which may be processed by DCL proteins as well (Molnár et al. 2005). In the context of antiviral defence, DCL4 and DCL2 play dominant and partially redundant roles. DCL4 preferentially processes dsRNAs into 21-nucleotide (nt) vsiRNAs, while DCL2 generates 22-nt vsiRNAs, particularly when DCL4 function is compromised (Blevins et al. 2006). These vsiRNAs guide the sequence-specific targeting of viral RNAs and are essential for effective antiviral immunity. Once produced, vsiRNAs are incorporated into Argonaute (AGO) proteins, forming the RNA-induced silencing complex (RISC) (Meister 2013). In *Arabidopsis thaliana,* ten different AGO proteins could be found, showing preferences for distinct classes of small RNAs (Meister 2013). AGO1 predominantly associates with 21-nt vsiRNAs, while AGO2 has been shown to preferentially bind 22-nt vsiRNAs and is often upregulated upon viral infection (Carbonell and Carrington 2015). The AGO-vsiRNA complex within RISC then mediates the cleavage or translational repression of complementary viral RNA targets, effectively reducing viral load (Brodersen et al. 2008). Secondary siRNAs originate from single-stranded viral transcripts that were already targeted by primary siRNAs before being converted into dsRNA by host RdRp. This step initiates an amplification loop that enhances the silencing response, known as transitivity, and is important to achieve silencing for viruses (Wang et al. 2010). Following local induction of RNA silencing, sRNAs can move approximately 10 to 15 adjacent cells through plasmodesmata, enabling short-range, cell-to-cell signal distribution (Dunoyer et al. 2013), before the sRNA signal needs to be enhanced again (Himber et al. 2003). For long-distance signalling, sRNAs are transported systemically via the phloem, allowing silencing to reach distant tissues where gene expression can be modulated, systemic immunity is activated or stress-related responses throughout the plant are coordinated (Kehr and Kragler 2018). The efficacy of RNA silencing is influenced by multiple factors, including the abundance, size distribution, and sequence specificity of vsiRNAs. Furthermore, many plant viruses encode viral suppressors of RNA silencing (VSRs) to counteract this defence (Kasschau and Carrington 1998). The functions of VSRs are highly diverse and strongly dependent on the particular virus species or family, as different viruses have evolved distinct strategies to interfere with different host RNA-silencing components (Burgyán and Havelda 2011). They can disrupt the functions of AGO and DCL proteins, inhibit vsiRNA production, or sequester duplex siRNAs, all of which facilitate successful viral infection (Li and Ding 2006). Examples of VSRs in plants are the helper component protease (HC-Pro) of potyviruses, which is known to bind small RNAs and thereby block RNA silencing before it can act (Shiboleth et al. 2007), or the P0 protein from poleroviruses, which prevents the association of AGO1 with siRNAs (Csorba et al. 2010). The balance between effective RNA silencing and viral suppression often determines whether infection can be stopped by the plant or progresses systemically. Another aspect of RNA silencing is the regulation of gene expression by messenger RNA (mRNA) degradation, facilitated by microRNA (miRNAs) (Fire et al. 1998). These miRNAs are encoded in MIRNA genes (Bartel 2004) and are often crucial for plant development, tissue differentiation and stress response (Ruiz-Ferrer and Voinnet 2009).

In recent years, the topical application of antiviral dsRNA sprays has gained increasing attention in research as a strategy to protect plants from subsequent viral infections. When applied exogenously, dsRNA can trigger sequence-specific degradation of complementary viral RNA. This was first demonstrated by Tenllado and Díaz-Ruíz (2001), who showed that dsRNA derived from pepper mild mottle virus (PMMoV) conferred protection against the virus in *Nicotiana tabacum* cv. Xanthi. While formulation strategies that enhance dsRNA stability and uptake, such as binding to carrier molecules (Mitter et al. 2017) or encapsulation in nanovesicles (Qiao et al. 2023), are a major focus of current research, the selection of antiviral target sequences within the viral genome remains important as well. The efficacy of RNA silencing depends not only on the delivery of dsRNA but also on whether the chosen viral region is both accessible to the plant RNAi machinery and functionally indispensable for the virus (Barroso et al. 2025; Luo and Chang 2004). Small RNA sequencing is a powerful tool in this context, as it reveals viral RNA regions that are preferentially processed into vsiRNAs by the host silencing machinery. These hotspots are likely to be structurally accessible and efficiently recognised by DCL enzymes, making them possible candidates for the design of effective antiviral dsRNAs (Mohamed et al. 2022).

Sugar beet (*Beta vulgaris* subsp. *vulgaris*) is an economically important crop that is susceptible to a range of viruses, including those grouped under the virus yellows (VY) complex, a major viral disease threat in Europe. This complex comprises several phloem-limited viruses that can have the potential to result in significant yield losses (Hossain et al. 2021). In order to identify effective antiviral target sites and to deepen the understanding of host–virus interactions at the molecular level, we analysed and compared the vsiRNA profiles generated during infection of *B. vulgaris*, the natural host, and the experimental host *Nicotiana benthamiana* with beet yellows virus (BYV, genus *Closterovirus*), beet mild yellowing virus (BMYV, genus *Polerovirus*), and beet mosaic virus (BtMV, genus *Potyvirus*), all transmitted by the aphid *Myzus persicae*. A sequence-by-sequence approach was used to compare vsiRNA sequences derived from one host plant with those from another. This comparison of vsiRNA abundances at the level of individual sequences revealed largely consistent patterns across both host species. Comparing these three different virus infections allowed us to detect differences and similarities in how viral RNAs were processed by the RNA silencing machinery in two different hosts, offering insights into the activity of DCL enzymes, recognition of viral RNA structures, and overall antiviral defence responses. These findings can support the targeted development of dsRNA-based control strategies, while also contributing to basic knowledge of RNA silencing mechanisms in plants. The BtMV vsiRNA profile had been characterised previously from the same dataset (Rahenbrock et al. 2025) and was here compared with the other viral infections of BMYV and BYV.

## 2 Materials and methods

### 2.1 Plant material, virus strains and virus transmission

*B. vulgaris* and *N. benthamiana* plants were grown under controlled greenhouse conditions with 24 °C/14 h light and 18 °C/10 h dark period. The isolates of BtMV (isolate from Fullbourn, UK and provided by DSMZ), BYV (supplied by Mark Stevens, BBRO UK), and BMYV (natural isolate from Hannover, Germany) were maintained on sugar beet cv. Vasco (SESVanderHave, Tienen, Belgium) to serve as inoculum for subsequent experiments. An aviruliferous *M. persicae* population (supplied by the Institute of Horticultural Production Systems, Department Phytomedicine, Leibniz University Hannover) was reared on healthy sugar beet cv. Vasco. For virus acquisition, aviruliferous aphids were allowed to feed on infected inoculum plants for two days (BtMV), five days (BYV), or seven days (BMYV) to ensure successful virus uptake. For virus inoculation, ten aphids carrying one of the viruses were transferred onto *N. benthamiana* or *B. vulgaris* plants at the second leaf stage. For the healthy controls, ten aphids from non-infected sugar beets were also transferred onto *N. benthamiana* or *B. vulgaris* to ensure that observed differences were not due to aphid feeding.

### 2.2 RNA isolation and sRNA sequencing by high-throughput sequencing (HTS)

For viral small RNA analysis, three independent virus-infected and one to three non-infected *N. benthamiana* and *B. vulgaris* leaf samples were sequenced. Sixteen days after BtMV and BYV infection or 22 days after BMYV infection, total RNA was isolated from symptomatic leaves using Direct-zol RNA Miniprep kit (Zymo Research, Irvine, USA) following the manufacturer’s instructions. Actual virus infection was validated with RT-qPCR, using the CFX96 Real-Time System (Bio-Rad, Feldkirchen, Germany). These time points were chosen because they consistently showed systemic infection across both host species, with the presence of characteristic systemic symptoms and positive RT-qPCR results.

RNA library preparation and sequencing were performed by Novogene Company (Cambridge, UK). Following quality control, 3′ and 5′ adaptors were ligated, and first-strand cDNA synthesis was carried out. PCR enrichment was used to generate double-stranded cDNA, and libraries containing fragments between 10 - 40 bp were selected for sequencing. Libraries were sequenced on the Illumina NovaSeq 6000 platform, generating at least 20 million reads per sample. Raw reads were filtered to exclude those with >50 % low-quality bases (quality score ≤ 5), >10 % ambiguous bases (N), 5′ primer contamination, absence of a 3′ primer or insert tag, presence of 3′ primer sequences, or homopolymeric sequences (poly A/T/G/C). Residual adapters were also removed.

### 2.3 Bioinformatic analysis

The remaining reads were subsequently mapped to the *N. benthamiana* reference genome Niben261 (Fernandez-Pozo et al. 2015) allowing one mismatch or to the *B. vulgaris* reference genome RefBeet 1.2.2 (Dohm et al. 2014) allowing one mismatch. The reads were also mapped to the corresponding virus genome: BtMV isolate DSMZ PV-1228 (Gen-Bank accession no MT815987), BMYV isolate IPP (accession no DQ132996) or BYV isolate P4–10F-W12 (accession no ON924234), allowing no mismatches. Mapping was done using CLC Genomics Workbench (version 24.0.3, Qiagen, Hilden, Germany). Read size, vsiRNA distribution along the viral genome, polarity, 5′-nt analysis were also performed using CLC. Visualisation was done by SigmaPlot (version 14.2). The vsiRNA comparison between both host plant species was calculated and visualised with Python (version 3.11). The log_2_-transformation of the counted data was realised by adding +1 to each value.

## 3 Results

### 3.1 BYV and BtMV-vsiRNA accumulate to high amounts, but BMYV-vsiRNAs do not

**Fig. 1:**
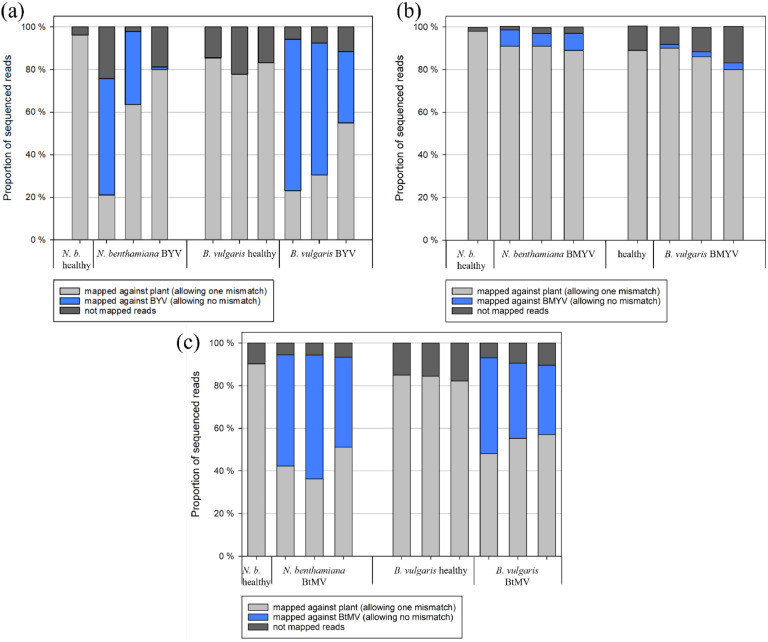
Ratio of small RNAs mapped against the host plants *Nicotiana benthamiana* or *Beta vulgaris* (allowing one mismatch) to small RNAs mapped against (a) beet yellows virus (BYV), (b) beet mild yellowing virus (BMYV) and (c) beet mosaic virus (BtMV) (allowing no mismatch) and not mapped reads. .

The effect of three virus infections, BYV, BMYV or BtMV, on sRNA accumulation was examined in *N. benthamiana* and *B. vulgaris* plants 14 days after infection (21 dpi for BMYV) with virus-loaded aphids. To assess the viral presence and distribution of sequencing small RNA reads, the proportion of reads mapping to host plant genomes and viral genomes was analysed (Fig. 1). In BYV-infected samples (Fig. 1a), a substantial proportion of small RNA reads mapped to the BYV genome in both *N. benthamiana* (34.25 % and 54.08 %) and *B. vulgaris* (33.51 %, 61.85 %, and 71.10 %). One BYV-infected *N. benthamiana* sample showed minimal BYV-derived small RNAs (1.22 %) and was left out of further analysis. Healthy control samples exhibited no reads mapping to the BYV genome. In the *N. benthamiana* healthy control, 96.18 % of reads mapped to the plant genome, with 3.81 % remaining unmapped. In BYV-infected *N. benthamiana* plants, 21.08 % to 80.04 % of reads aligned with the plant genome, while 2.4 % to 24.12 % of reads remained unmapped. In the *B. vulgaris* healthy controls, between 77.78 % and 85.42 % of the total reads could be mapped against the host genome, leaving 14.37 % to 22.22 % of reads unmapped. In BYV-infected *B. vulgaris* samples, 23.19 %, 30.58 % and 54.94 %, respectively, of total reads could be aligned to the host genome, while 5.71 % to 11.55 % remained unmapped. For BMYV-infected plants (Fig. 1b), only a small fraction of reads mapped to the BMYV genome. In sugar beets, 1.76 % to 3.1 % of reads aligned with the viral genome, while in *N. benthamiana*, slightly higher proportions were observed (5.95 %, 7.61 % and 7.97 %, respectively). The majority of small RNAs were host-derived, with 89.08 % to 91.30 % of reads mapping to the *N. benthamiana* genome, and 79.79 % to 90.00 % mapping to the *B. vulgaris* genome. In healthy controls, 98.19 % and 88.55 % of reads mapped to the *N. benthamiana* and *B. vulgaris* genomes, respectively. Unmapped reads constituted <3 % in all *N. benthamiana* samples and between 8.24 % and 17.10 % in sugar beet samples. BtMV-infected samples (Fig. 1c) showed a high proportion of virus-mapped reads, ranging from 43.35 % to 57.91 % in *N. benthamiana*, and from 32.44 % to 44.97 % in *B. vulgaris*. In the healthy *N. benthamiana* sample, 90.48 % of reads aligned to the host genome, with 9.51 % being unmapped. In BtMV-infected *N. benthamiana*, 36.44 % to 51.84 % of reads mapped to the plant genome, with around 6 % being unmapped. In *B. vulgaris* samples infected with BtMV, 48.08 % to 57.14 % of reads mapped to the host genome, with 6.95 % to 10.42 % being unmapped. In the healthy sugar beet control, around 84 % of small RNAs mapped to the plant genome (full table found in table S1).

### 3.2 Differential size profile of host sRNAs and vsiRNAs

**Fig. 2:**
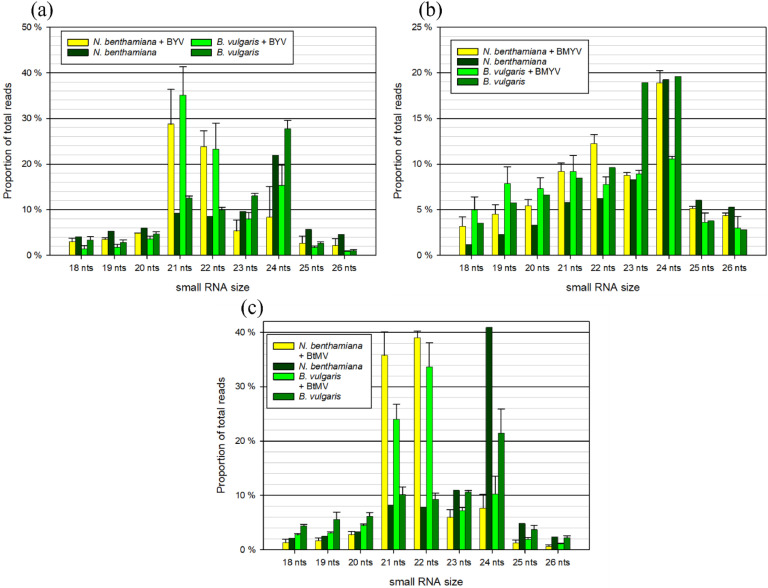
Size distribution of total small RNAs in the virus-infected and healthy host plants *Nicotiana benthamiana* and *Beta vulgaris* libraries for (a) beet yellows virus (BYV), (b) beet mild yellowing virus (BMYV) and (c) beet mosaic virus (BtMV). For visualization, only reads of size 18 - 26 bp are shown. Standard error is included, when three individual samples were sequenced.

The size distribution of sRNAs mapped to the plant genomes was analysed in *N. benthamiana* and *B. vulgaris* under BYV, BMYV, and BtMV infection, respectively, alongside healthy controls (Fig. 2). Across all conditions, sRNAs ranging from 18 to 26 nt in length were analysed, with the highest proportions generally observed in the 21 - 24 nt range. In both *N. benthamiana* and *B. vulgaris* plants, BYV-infected samples (Fig. 2a) showed similar patterns, with the 21 nt class reaching up to approximately 30 - 35 % of plant-mapped reads, followed by 22 nt sRNAs, with 20 - 30 %. In the healthy samples, the 24 nt sRNA was the predominant size in both host plants with around 20 - 30 % abundance. In BMYV-infected samples (Fig. 2b), 24 nt sRNAs constituted the largest proportion of plant-mapped reads across all samples, with almost 20 %. This peak was particularly pronounced in *N. benthamiana*, while in BMYV-infected *B. vulgaris* only 10 % of the total plant-mapped sRNAs belonged to the 24-nt class. Other prominent size classes included 21 nt, 22 nt, and 23 nt sRNAs. The 23 nt sRNA class showed a notably higher abundance in the healthy *B. vulgaris* sample, where its proportion was approximately twice that observed in the infected variants (Fig. 2b). In BtMV-infected samples (Fig. 2c), the distribution of plant-mapped sRNAs varied more across conditions. In BtMV-infected *N. benthamiana* and *B. vulgaris*, the 22 nt class represented the largest fraction of plant-mapped reads, with proportions reaching up to 35 - 40 %, followed by 21 nt vsiRNAs with 25 - 35 %. In healthy *N. benthamiana* and *B. vulgaris*, the 24 nt class was the most prominent class with 20 % to 40 % of plant-mapped reads belonging to this class (Fig. 2c).

**Fig. 3:**
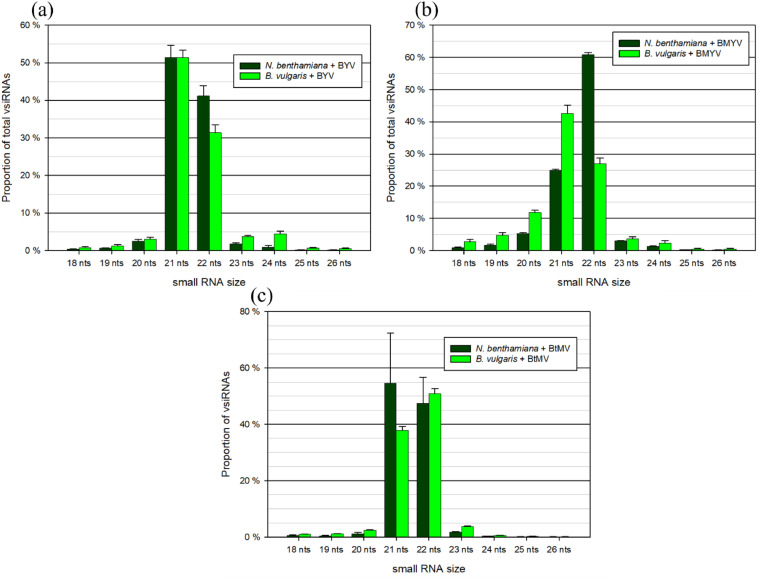
Size distribution of virus-derived small RNAs in the virus-infected host plants *Nicotiana benthamiana* and *Beta vulgaris* libraries for (a) beet yellows virus (BYV), (b) beet mild yellowing virus (BMYV) and (c) beet mosaic virus (BtMV). For visualization, only reads of size 18 - 26 bp are shown. Standard error is included, when three individual samples were sequenced.

The size distribution of vsiRNAs was analysed in *N. benthamiana* and *B. vulgaris* infected with BtMV, BYV, and BMYV. Across all virus infections, vsiRNAs predominantly fell within the 21 - 22 nt size range, with minor representation from other length classes. In BYV-infected samples (Fig. 3a), 21 nt vsiRNAs constituted the most abundant class in both host plants, comprising over 50 % of total vsiRNAs. In BMYV-infected samples (Fig. 3b), 22 nt vsiRNAs were the most prominent class in *N. benthamiana*, while in *B. vulgaris,* the most prominent class was 21 nt vsiRNAs, which reached over 40 % of the total vsiRNAs. The corresponding other class represented the second-highest proportion. In BtMV-infected *N. benthamiana*, 21 nt vsiRNAs accounted for the highest proportion, reaching 60 % of total vsiRNAs, followed by 22 nt sRNAs at approximately 30 %. The opposite pattern was observed in *B. vulgaris*, with 21 nt vsiRNAs being most abundant, followed by 22 nt (Fig. 3c). Across all virus and plant species, other size classes, including 18 - 20 nt and 23 - 26 nt, were present at low levels.

### 3.3 The vsiRNA profile is virus-dependent, not host-dependent

**Fig. 4:**
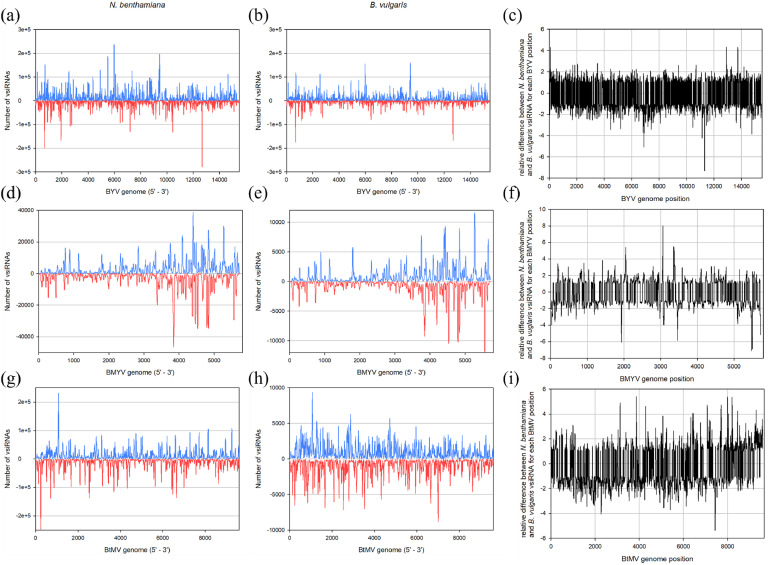
Mapping of (*a* + *b*) beet yellows virus (BYV), (*d* + *e*) beet mild yellowing virus (BMYV) and (*g* + *h*) beet mosaic virus (BtMV) derived small RNAs from infected *Nicotiana benthamiana* (a, d, g) and *Beta vulgaris* (b, e, h) to the reference virus genome allowing no mismatch. Positive numbers refer to sense, negative numbers to antisense viral sRNAs. The relative difference of BYV (c), BMYV (f) and BtMV (i) derived vsiRNAs between *N. benthamiana* and *B. vulgaris* was calculated for each genome position. Positive values indicate higher vsiRNA abundance in *N. benthamiana*, negative values indicate higher abundance in *B. vulgaris*.

For each sample, the viral small RNA sequences were collected and mapped to the corresponding virus genome to create a vsiRNA profile (Fig. 4). To better compare vsiRNA peak positions between hosts, the ratio of relative vsiRNA abundance at each position along the viral genome was calculated between *N. benthamiana* and *B. vulgaris* (Fig. 4c, F, i). The other vsiRNA profiles are shown in supplementary Figs. S1, S2 and S3.

The BYV-derived sRNAs (Fig. 4a+*b*) were evenly distributed across the whole viral genome length, with major sense peaks found at positions 680, 6000 and 9400 in both plant species. From the antiviral strand, peaks were found at positions 680 and 12,700. In *N. benthamiana,* these peaks reached up to 28,000 counts, whereas in *B. vulgaris* the maximum was at 18,000 counts. However, in direct comparison, no major differences could be recognised, except for positions 6900, 11,300, 12,900 and 13,700 (Fig. 4c). In BMYV-infected *N. benthamiana* and *B. vulgaris* (Fig. 4d+*e*), vsiRNAs predominantly accumulated in the 3′ half of the BMYV genome, forming major peaks similar for both strand polarity. Similar peaks were observed in *B. vulgaris*, although increased peaks could be located between positions 5000 and 6000. A comparison of the host-specific vsiRNA profiles revealed only minor differences, with increased vsiRNA accumulation in *N. benthamiana* at positions 2040, 3060, and 3350, and in *B. vulgaris* at positions 1920, 3460, and 5470 (Fig. 4f). In the *N. benthamiana* BtMV vsiRNA profile (Fig. 4g+*h*), several distinct hotspots were observed, distributed across the whole genome. The two highest peaks located near the 5´-end in the P1 region, with one peak positioned around nucleotide 200 of antisense origin and one peak around nucleotide 1000 on the viral sense strand. In contrast, the *B. vulgaris* profile exhibited a higher number of vsiRNA hotspots, although these were generally of lower intensity. In the comparison of all BtMV profiles (Fig. 4i), differences could be observed at certain positions. The regions around nucleotide 3100, 3900 and between 7100 and 8500 were particularly noteworthy, where more pronounced peaks could be identified in *N. benthamiana*. Conversely, at position 7450, the number of detected vsiRNAs was found to be five times higher in infected *B. vulgaris*. The most notable vsiRNA-profile difference between both plant species could be found after BtMV infection.

**Fig. 5:**
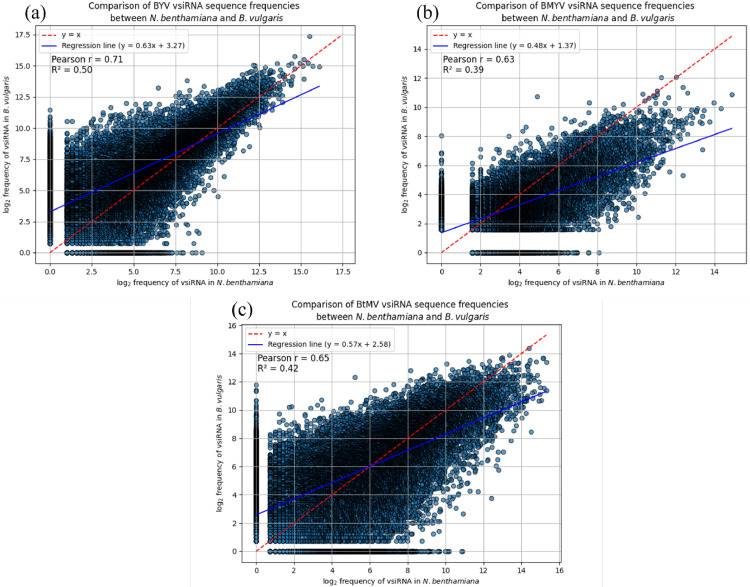
Comparison of (a) beet yellows virus (BYV), (b) beet mild yellowing virus (BMYV) and (c) beet mosaic virus (BtMV) vsiRNA sequence frequencies between *Nicotiana benthamiana* and *Beta vulgaris*. Each dot represents the log_2_-counts of a small RNA sequence in *N. benthamiana* and *B. vulgaris*.

The vsiRNAs were compared based on their sequence and added together, so that the absolute number of each vsiRNA sequence was obtained. These counts were normalised to compare the lists between the two different host plants in a scatter plot (Fig. 5). Each dot represents one vsiRNA sequence and its abundance in *N. benthamiana* and *B. vulgaris* after the corresponding viral infection. After infection with one of the three viruses, some vsiRNAs were detected exclusively in either *N. benthamiana* or *B. vulgaris*, but the majority were shared between both host plants. Specifically, 70 % of the 32,500 unique BYV-derived vsiRNAs were found in both species. Similarly, 75 % of the 21,000 unique vsiRNAs from BMYV and 76 % of the 28,000 unique vsiRNAs from BtMV were detected in both *N. benthamiana* and *B. vulgaris*. For BYV, the most abundant vsiRNA in *N. benthamiana* accounted for 0.75 % of total reads, whereas in *B. vulgaris* it reached 0.8 %. Similar patterns were observed for BMYV and BtMV, where a few highly abundant vsiRNAs accounted for a disproportionate number of total reads. Comparative analysis of vsiRNA abundance revealed a moderate to strong linear correlation between *N. benthamiana* and *B. vulgaris*, with Pearson correlation coefficients ranging from 0.63 (BMYV) to 0.71 (BYV). This indicates that highly abundant vsiRNAs in one host were typically also highly abundant in the other.

### 3.4 Sense, antisense and 5′-nt distribution of vsiRNAs

**Fig. 6:**
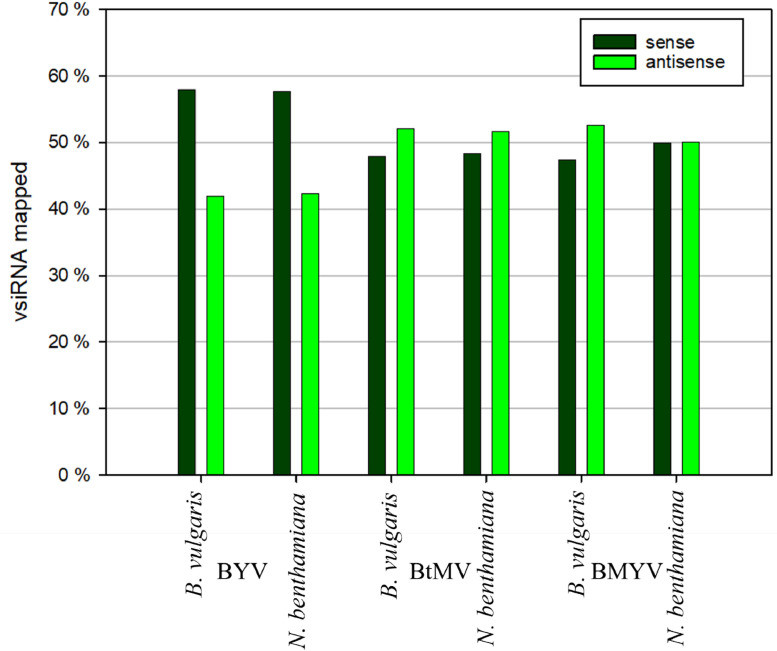
Beet yellows virus (BYV), beet mild yellowing virus (BMYV) and beet mosaic virus (BtMV) vsiRNA strand polarity from infected *Nicotiana benthamiana* and *Beta vulgaris*.

Analysis of vsiRNA strand polarity revealed differences in the proportion of sense and antisense reads (Fig. 6). For BYV, vsiRNA populations in both host plants were biased toward the sense strand, with approximately 58 % of vsiRNAs mapping in the sense orientation. In contrast, infection with BMYV and BtMV displayed a balanced or antisense-skewed distribution. In both host plants infected with BtMV, antisense vsiRNAs represented around 52 % of total vsiRNAs, slightly exceeding the sense-derived vsiRNAs. The BMYV-infected sugar beets showed an equal distribution of sense and antisense reads, whereas *N. benthamiana* exhibited a predominance of antisense vsiRNAs with 53 %.

**Fig. 7:**
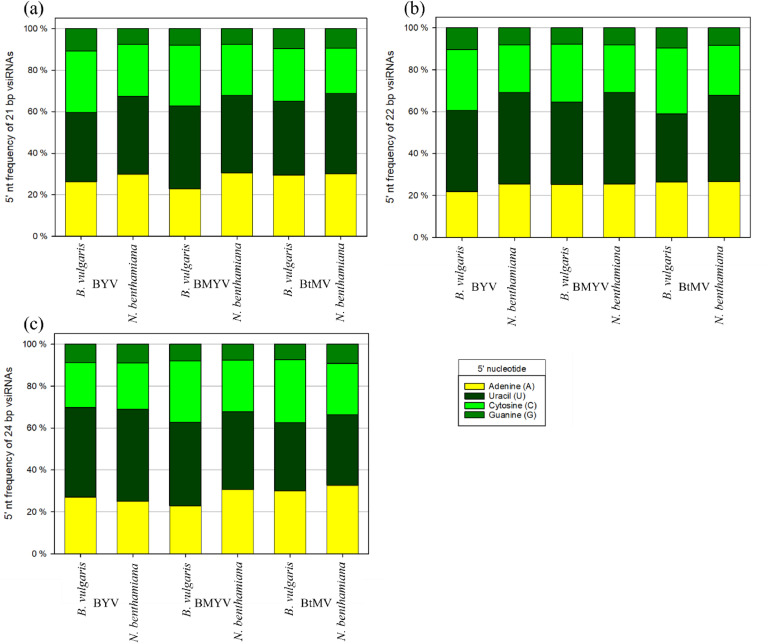
5′ terminal nucleotide identity of beet yellows virus (BYV), beet mild yellowing virus (BMYV) and beet mosaic virus (BtMV) vsiRNA based on their sequence length (a) 21 bp, (b) 22 bp and (c) 24 bp in *Nicotiana benthamiana* and *Beta vulgaris*.

The identity of the 5′ terminal nucleotide of vsiRNAs is an important determinant for their sorting into specific AGO proteins (Fig. 7). A strong uracil (U) bias was seen in 21- and 22-nt vsiRNAs across all virus-host combinations (∼39 - 44 %). Cytosine (C) was more frequent in *B. vulgaris*, while adenine (A) was more prominent in *N. benthamiana*. Guanine (G) remained consistently low (∼7 - 10 %). For 24-nt vsiRNAs, U was dominant for BYV and BMYV, whereas BtMV showed a more even distribution of A, U, and C (∼30 %), while G stayed lowest in all cases.

## 4 Discussion

This study provides a comprehensive comparison of virus-derived small interfering RNAs from the aphid-transmitted sugar beet-infecting viruses BtMV, BYV and BMYV in the two different host plants *B. vulgaris* and *N. benthamiana*. The analysis revealed clear differences in the accumulation levels, size distribution, genomic origin and sequence features of vsiRNAs between the different virus species. Interestingly, only minor differences in the RNAi processing patterns were observed between the infection of the natural host *B. vulgaris* and the experimental host *N. benthamiana*, indicating that vsiRNA profiles are primarily virus dependent, not host dependent. Virus-specific factors, including the activity of viral suppressors of RNA silencing, genome accessibility, and replication strategy, primarily determine vsiRNA abundance and processing. Even though *N. benthamiana* carries a natural variant of an RdRp and is highly susceptible to viruses (Yang et al. 2004), the distinct vsiRNA profiles observed for BYV, BMYV, and BtMV indicate that viral characteristics are the main drivers of RNAi efficacy. This study can also be used to develop dsRNA-sprays against the different viruses.

Among the three viruses, BYV and BtMV triggered high levels of vsiRNA accumulation, with over 30 - 70 % of total small RNA reads mapping to their respective genomes in infected samples. Similar numbers were detected for the potyvirus potato virus Y (PVY) (Shidore et al. 2020; Prigigallo et al. 2019). This indicates that both virus genomes are efficiently recognised and processed by the host RNA silencing machinery. In contrast, BMYV consistently produced markedly lower vsiRNA levels, particularly in sugar beet, even though virus titres were high in all samples (data not shown). Silva et al. (2011) and Zhang et al. (2017) also discovered that about 5 - 10 % of total sRNAs belonged to the respective polerovirus. This suggests a reduced engagement of BMYV with the RNA silencing pathway or the presence of potent viral suppressors that inhibit vsiRNA biogenesis. BMYV encodes the P0 protein, a well-characterised VSR that targets the AGO1 protein, prevents the assembly of the RISC complex and leads to degradation of AGO1 (Bortolamiol et al. 2007; Csorba et al. 2010). Furthermore, P0 likely also suppresses secondary siRNA production, as this mechanism depends on AGO proteins (Baumberger et al. 2007). This may explain the limited vsiRNA accumulation observed in BMYV-infected plants despite high viral replication levels. Another contributing factor could be BMYV’s strict phloem limitation, which confines the virus to fewer infected cells. Moreover, the genome structure of BMYV may be less accessible to Dicer processing, further reducing vsiRNA production. In contrast, BtMV encodes HC-Pro, a multifunctional protein known to suppress RNA silencing by especially binding and sequestering small RNAs (Maia and Bernardi 1996). The high levels of vsiRNAs observed in BtMV-infected samples suggest that HC-Pro only binds vsiRNAs and thereby hinders their antiviral function but not degrade AGO1 or block primary vsiRNA production (Lakatos et al. 2006). BYV encodes at least one distinct VSR, p21, which binds double-stranded sRNAs with high affinity, preventing their incorporation into AGO-containing complexes (Chapman et al. 2004). Like HC-Pro, p21 does not target AGO1 for degradation but instead inhibits silencing binding sRNAs directly (Chapman et al. 2004; Reed et al. 2003). The observed differences in vsiRNA abundance are thus best explained by virus-specific factors, such as genome structure, replication strategy or the function of their VSRs. Overall, BYV and BtMV might be more effectively processed into vsiRNAs, while BMYV evades or suppresses this pathway more successfully, potentially limiting the effectiveness of RNAi-based antiviral approaches targeting BMYV.

The size profile of vsiRNAs across all virus infections revealed a clear dominance of 21- and 22-nucleotide classes, which are typical products of the Dicer-like enzymes DCL4 and DCL2, respectively (Blevins et al. 2006). This predominance has been reported for many other plant viruses like tobacco mosaic virus (TMV) (Qi et al. 2009), potato virus X (PVY) (Donaire et al. 2009) or bamboo mosaic virus (BaMV) (Lin et al. 2010).

The distribution of vsiRNAs along viral genomes has been shown to reveal virus-specific accumulation patterns. Previous studies have indicated that these patterns do not differ significantly between different host plants (Margaria et al. 2016; Mitter et al. 2017). In the case of BtMV, vsiRNAs were relatively evenly distributed, with several distinct hotspots, as already described by Shidore et al. (2020). Similarly, BYV displays several distinct hotspots across its genome, which appear to contrast with previously published vsiRNA profiles of other closteroviruses, where a strong bias toward the 3′ end was typically observed (He et al. 2015; Ruiz-Ruiz et al. 2011; Wang et al. 2020). Because the vsiRNA pattern in our study was consistent across replicates, the differences from previous reports are most likely explained by unique features of the BYV genome compared to other closteroviruses (Dolja et al. 2006), or to variation in the stage of infection affecting vsiRNA processing, since in our study only one time point was sampled and analysed. On the other hand, BMYV-derived vsiRNAs showed a strong bias toward the 3′ end of the genome, possibly due to the strong accumulation of viral sgRNAs, similar to the cotton leafroll dwarf polerovirus (CLRDV) (Silva et al. 2011). Although BYV produce seven known sgRNAs (Dolja 2003), this is not clearly reflected in the vsiRNA profile, suggesting that sgRNA-derived dsRNA structures may not significantly contribute to Dicer processing. Another explanation for this could be that the different sgRNAs are not necessarily expressed at substantially higher levels than the genomic RNA and thus may not contribute disproportionately to vsiRNA generation (Donaire et al. 2009; Hagiwara et al. 1999; Qi et al. 2009).

Despite small differences in total vsiRNA abundance and genomic distribution between *N. benthamiana* and *B. vulgaris*, the most abundant vsiRNA sequences were largely conserved between the two host species. Approximately 70 - 76 % of vsiRNA species were shared for each virus, and their relative abundances were moderately to strongly correlated (Pearson’s *r* = 0.63 - 0.71). This indicates that, while host genotype may influence overall vsiRNA yield and hotspot prominence to some extent, the viral genome is the primary determinant of vsiRNA biogenesis (Mitter et al. 2013). The broadly conserved nature of the RNA silencing machinery across plant species (Baulcombe 2004) allows for comparable processing of viral RNA, although subtle differences in infection severity, tissue type, viral load, and RNA structure may modulate the efficiency and pattern of vsiRNA production (Li et al. 2017). By directly comparing vsiRNA sequences derived from the same virus species in two different hosts, this study provides a detailed view of host-specific vsiRNA processing and reveals novel differences not apparent from coverage profiles alone. Furthermore, vsiRNAs, which are present in one host plant but not in the other, may indicate host-specific interactions with the virus. They could indicate different host defences or an alternative vsiRNA biogenesis, or they could be cross-kingdom mobile RNAs, if they have complementarity to host genes, with the potential to regulate gene expression in the corresponding plant and facilitate successful infection (Liu et al. 2021; Ramesh et al. 2020).

The observed preference for uracil at the 5′ terminal of vsiRNAs is consistent with known AGO1 loading specificity. AGO1 preferentially binds 21–22 nt small RNAs beginning with a 5′ uracil, which supports their role in antiviral defence (Mi et al. 2008). Since AGO loading and silencing efficiency partly depend on the 5′ nucleotide, designing dsRNA sprays that favour U at the 5′ vsiRNA end might increase the chance of vsiRNAs being sorted into AGO1, enhancing antiviral efficiency. No experiments were conducted to investigate whether the sampling time point influences vsiRNA profiles.

All the *in situ* results can be used to develop suitable target regions for dsRNA sprays against BMYV, BYV and BtMV. From an applied perspective, patterns are highly relevant for the design of dsRNA constructs for spray-induced gene silencing. Targeting regions that naturally give rise to abundant vsiRNAs may improve the efficiency of RNAi-based control strategies by aligning with the host’s inherent Dicer processing preferences (Mohamed et al. 2022). The previous publication, also based on these data, demonstrated that exogenously applied dsRNA derived from P1 and NIb regions could induce resistance against BtMV under controlled conditions (Rahenbrock et al. 2025). In the case of BYV and BMYV, targeting the 5′ region of the viral genome may be particularly advantageous. For BYV, a dsRNA spray targeting regions around nucleotides 700 or 5960, both within ORF1a, could be effective, as these sites correspond to prominent vsiRNA peaks in both host plants and fall within the genomic RNA. Because both regions encode part of the RdRp, their silencing would inhibit viral replication, which is indispensable for genome amplification and the production of subgenomic RNAs required for viral protein translation (Buck 1996). For BMYV, regions around nucleotides 280 or 1800 could be targeted, with position 280 additionally affecting the VSR P0, potentially enhancing antiviral activity by disabling the virus’s RNAi suppression mechanism. Silencing both RdRp and VSR proteins has been shown to be particularly effective as RNAi targets across different virus families (Kaldis et al. 2018; Konakalla et al. 2021; Kweon et al. 2022), as RNAi constructs targeting RdRp provide stronger and more durable resistance than those directed against structural proteins (Villegas-Estrada et al. 2022), while silencing P0 can restore the host’s RNA silencing defence. The effectiveness of these dsRNA treatments, however, must be validated under greenhouse conditions using virus-loaded aphids.

The findings of this study provide valuable insights for the development of targeted RNAi-based antiviral strategies, particularly using exogenously applied dsRNA. The distinct vsiRNA profiles observed for each virus, including differences in accumulation levels, size classes, and hotspot regions, offer a blueprint for selecting the most effective viral sequences for silencing. By designing dsRNA constructs that mimic naturally abundant vsiRNA-generating regions, it is possible to enhance Dicer processing efficiency and trigger stronger antiviral responses, potentially offering a sustainable and adaptable tool for controlling viral diseases in crops. However, the effectiveness of such RNAi-based approaches may be influenced by virus-specific factors, including the activity of VSRs, genome accessibility, and replication strategy. For instance, it appears that BMYV’s P0 protein strongly inhibits RNA silencing and limits vsiRNA accumulation, suggesting that some viruses may evade or suppress host RNAi more effectively than others, which should be considered when designing targeted dsRNA constructs.

## Funding

This research did not receive any specific grant from funding agencies in the public, commercial, or not-for-profit sectors.

## CRediT authorship contribution statement

**Dennis Rahenbrock:** Writing – original draft, Visualization, Software, Investigation, Formal analysis, Conceptualization. **Mark Varrelmann:** Writing – review & editing, Supervision, Conceptualization.

## Declaration of competing interest

The authors declare that the research was conducted in the absence of any commercial or financial relationships that could have appeared to influence the work reported in this paper.

## Data Availability

The data that support the findings of this study are available from the corresponding author upon reasonable request.

## Appendix Supplementary materials

Image, application 1

Image, application 2

Image, application 3

Image, application 4

Image, application 5
