# Supplementary figures and images for: Comparative small RNA profiles of beet mosaic virus (BtMV), beet mild yellowing virus (BMYV) and beet yellows virus (BYV) infected Nicotiana benthamiana and Beta vulgaris
Source: Virus Res. 2025 Oct 10;361:199640. doi: 10.1016/j.virusres.2025.199640 (PMC12550339; doi:10.1016/j.virusres.2025.199640)

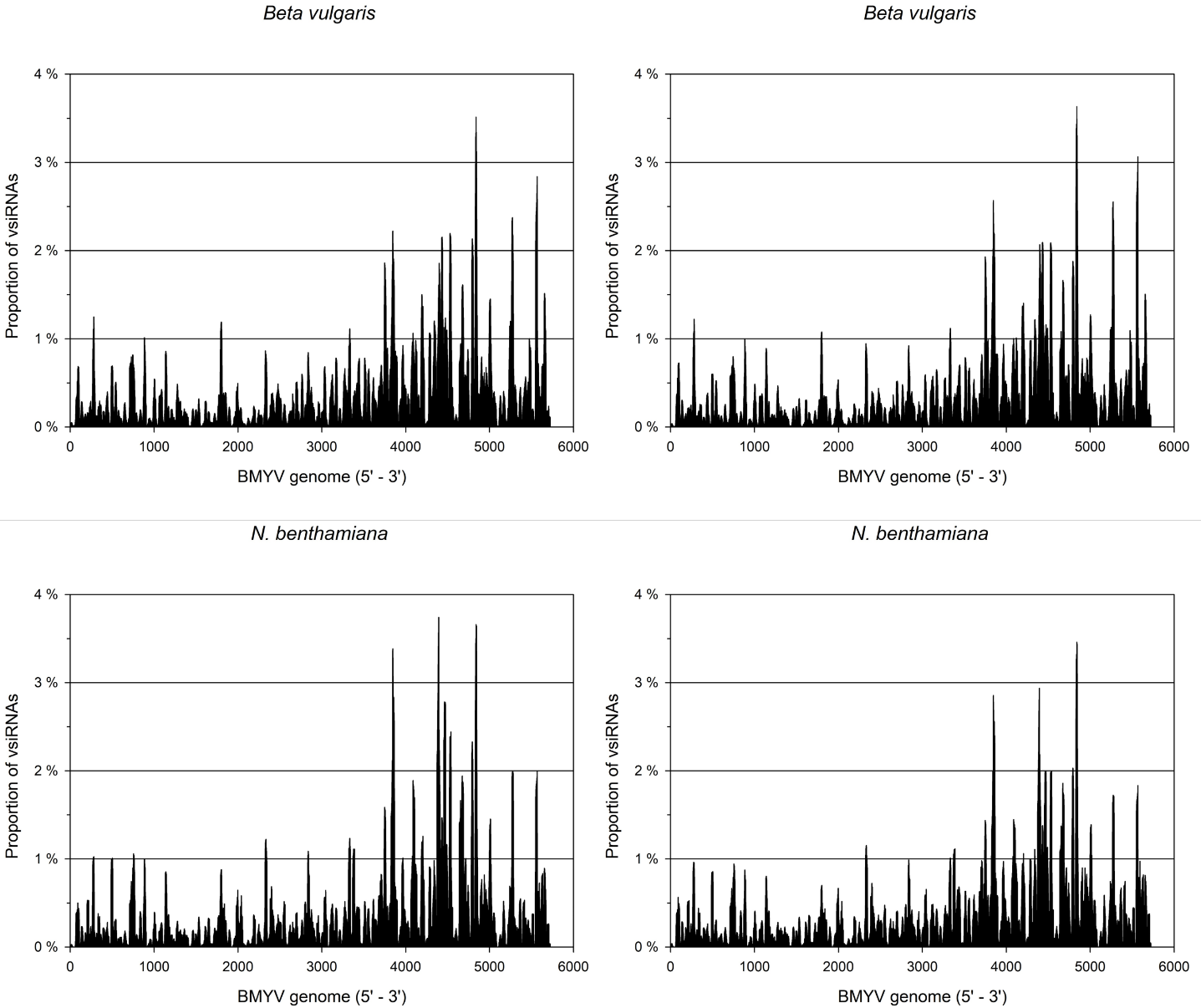

Supplement: Supplementary file 1 [file mmc1.docx]

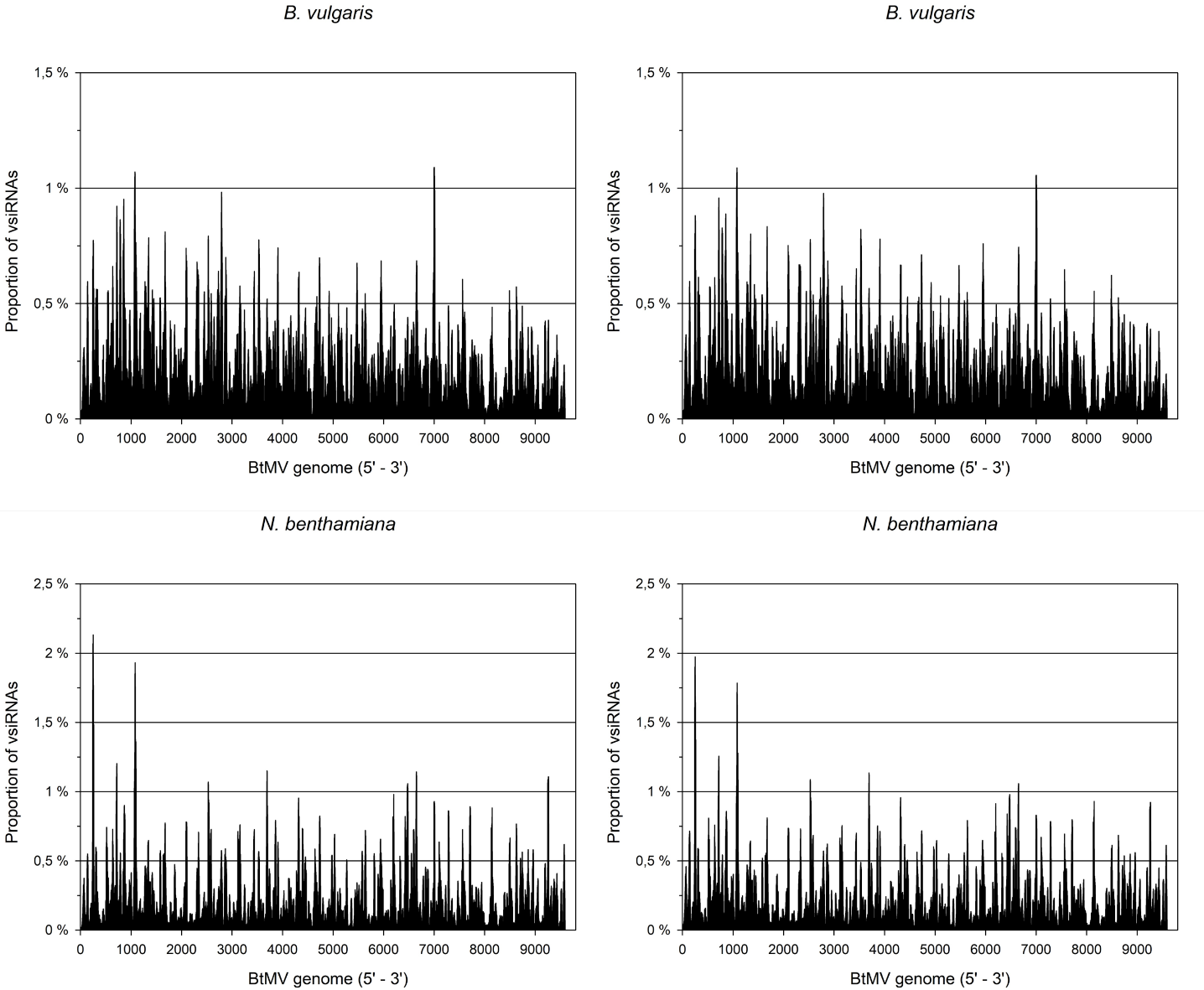

Supplement: Supplementary file 2 [file mmc2.docx]

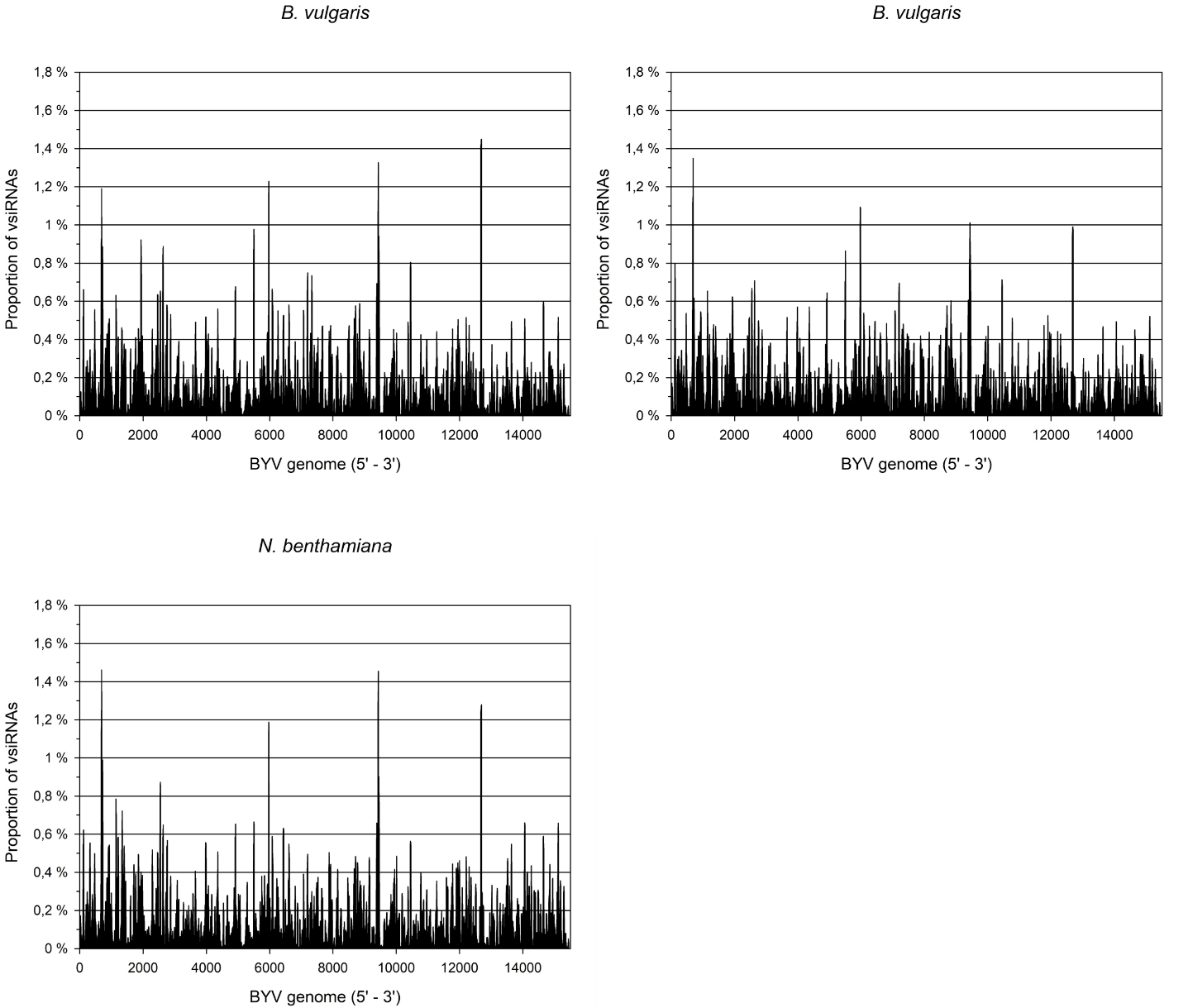

Supplement: Supplementary file 3 [file mmc3.docx]
