# Supplementary material for: Comparative small RNA profiles of beet mosaic virus (BtMV), beet mild yellowing virus (BMYV) and beet yellows virus (BYV) infected Nicotiana benthamiana and Beta vulgaris
Source: Virus Res. 2025 Oct 10;361:199640. doi: 10.1016/j.virusres.2025.199640 (PMC12550339; doi:10.1016/j.virusres.2025.199640)
Supplement: Supplementary file 4 [file mmc4.docx]

Figure, table and supplementary legends:

Supplementary table 1: Overview of small RNA sequencing data for beet yellows virus (BYV), beet mild yellowing virus (BMYV) and beet mosaic virus (BtMV) infected and healthy *Beta vulgaris* and *Nicotiana benthamiana*. Reads were mapped against genome of the plants allowing one mismatch and mapped against the virus allowing no mismatch. Sequencing was usually done in triplicates (rep 1 - 3).

Supplementary figure S1: Mapping of remaining beet mild yellowing virus (BMYV) derived small RNAs from infected *Nicotiana benthamiana* and *Beta vulgaris* to the reference virus genome allowing no mismatch.

Supplementary figure S2: Mapping of remaining beet mosaic virus (BtMV) derived small RNAs from infected *Nicotiana benthamiana* and *Beta vulgaris* to the reference virus genome allowing no mismatch.

Supplementary figure S3: Mapping of remaining beet yellows virus (BYV) derived small RNAs from infected *Nicotiana benthamiana* and *Beta vulgaris* to the reference virus genome allowing no mismatch.
